# Sociocultural drivers of mycological knowledge: insights from Wixarika and Mestizo groups in western Mexico

**DOI:** 10.1186/s13002-022-00564-2

**Published:** 2022-11-18

**Authors:** Mara Ximena Haro-Luna, José Blancas Vázquez, Felipe Ruan-Soto, Laura Guzmán-Dávalos

**Affiliations:** 1 Department of Botany and Zoology, University of Guadalajara, Apdo. Postal 1-139, 45147 Zapopan, JAL Mexico; 2 Centro de Investigación en Biodiversidad y Conservación, Universidad Autónoma del Estado de Morelos, Cuernavaca, Mexico; 3 Instituto de Ciencias Biológicas, Universidad de Ciencias y Artes de Chiapas, Tuxtla Gutiérrez, Mexico

**Keywords:** Cultural significance, Edible mushrooms, Ethnomycology, Huichol, Mushrooms, Toxic mushrooms, Wild mushrooms

## Abstract

**Background:**

Traditional mycological knowledge (TMK) is complex, not distributed equally among the entire population, and constantly adapting to current social situations. There are sociocultural factors that could influence the fact that some people retain a greater wealth of knowledge, for instance, cultural affiliation, migration, occupation, level of schooling, and person's age.

**Methods:**

We analyze the distribution of the TMK based on sociocultural variables and 12 indicators to quantify the TMK based on a literature review. We chose two sites where there was a Wixarika and Mestizo population with records of use and consumption of wild mushrooms. In each site, 150 semi-structured interviews were conducted. The format of the semi-structured interviews was made up of sociocultural questions plus 12 questions corresponding to each of the indicators. With the data obtained, we performed linear regression tests and principal components analysis (PCA); furthermore, the significance of the groupings obtained by PCA was tested with a discriminant function analysis.

**Results:**

We find that TMK was determined by the cultural group to which a person belongs. Contrary to what was expected, age and formal schooling did not influence people's level of knowledge. Likewise, migration and occupation were not determining factors either, although in some specific cases they did influence the differences in knowledge about mushrooms between people. The indicators that most helped to differentiate between the Wixarika people, and the Mestizos were knowledge of the nutritional contribution, propagation methods, and knowledge about toxic mushrooms.

**Conclusions:**

In general, sociocultural differences did not affect the transmission of the TMK due to the valorization of this knowledge among the young generations and the maintenance of the use of wild resources. Specifically, the Wixaritari had and preserved a greater TMK thanks to their pride in their cultural identity, which had allowed them to adapt to modernity while preserving their traditions and knowledge. On the other hand, the Mestizos increasingly disused wild resources due to urbanization. The indicators proposed here provided a good tool to quantify TMK; however, to replicate the study in other sites it is necessary to adapt the indicators to the context of the place.

## Background

The knowledge, practices, and beliefs about the relationship between living beings, including humans, and their environment constitute traditional ecological knowledge (TEK), which is transmitted from one generation to another [1]. This knowledge is related to the culture, language, and worldview of a human group [2, 3], explaining the world, nature, functioning of society, history, rituals, and social organization, as well as interactions within surrounding environment [4]. It is the product of the accumulation of experience and practices in resource use in a particular area throughout history [5, 6]. This accumulation of knowledge is considered culturally and environmentally specific because it allowed the people to develop within their territories, using the local resources [6].

Within the TEK is the traditional mycological knowledge (TMK), which is the set of beliefs, notions, and practices related to fungal diversity that help humans to understand the nature of fungi [7]. It is the product of years of observation and trial and error processes resulting from people's relationship with the available funga. However, knowledge is not static; environmental, social, and historical changes modify this accumulation of wisdom to adapt it to the present reality of each society, or in other cases, it can cause the replacement of local knowledge by that of hegemonic cultures, such as western modernity [5, 8]. Likewise, this knowledge is neither acquired nor distributed equitably throughout the population of the same culture [9]. In general, knowledge is acquired gradually throughout the life of individuals and is influenced by different social factors such as ethnic affiliation, migration, occupation, level of education, and age [5, 9–13]. Under these factors, people create mental models and possess a corpse of knowledge adapted to their context [14, 15].

It has been shown that Indigenous people know a large number of fungal species [16, 17]. In regions where different cultural groups coexist, e.g., Mestizos (mixture of Amerindian and European ancestors) and people belonging to an Indigenous group, the latter generally know a greater diversity of fungi, their ecology, phenology, uses, myths, recipes, and have a fine taxonomic knowledge to determine species (whether they use them or not). Precisely, this was observed by us [18] in the northern zone of Jalisco, Mexico, where the Wixaritari (an indigenous group from western Mexico, also known as Huichol) and the Mestizos have lived together for approximately 200 years. Both cultural groups have contact with the same biota, take advantage of the wild resources of the ecosystems that surround them, and exist a sustained trade between both cultures. Due to the conditions of poverty that prevail in these communities, for people of both cultural groups, wild edible mushrooms are a highly valued resource. They consider mushrooms as a delicious and nutritious food that they can only access for a short period of time during the rainy season. Despite this, it is the Wixaritari who know and use a greater number of species and who know in what type of vegetation each species can be found, recognize their function as degraders and as important actors in completing the cycle of life and death of organisms. Additionally, within their worldview, the Wixaritari consider that toxic mushrooms are the property of God and not simple harmful elements, as is the appreciation of the Mestizos [18].

In other matters, migration is another factor that can influence TEK modification. It has been reported that migrants adapt to the new environment by substituting the plants they used in their place of origin for those that they can find in the new site or by seeking to obtain the same species by import [19]. Thus, it has been seen that ethnobotanical knowledge, for example, has increased by incorporating new species and uses of other plants [20]. It is also the case that, although the migration is momentary, upon returning to their place of origin, people might replace the knowledge and use of local wild resources with global knowledge and prefer to use widely distributed products that they can buy [21–23]. In the case of the use of fungi, by not finding the species that migrants recognize, their knowledge, traditions, and management related to them could be lost, interfering with the transmission of ethnomycological knowledge [24, 25]. However, this knowledge may be adapted to the new funga with which people have contact [26, 27].

On the other hand, societies that base their economy on activities related to agricultural activities have a greater knowledge of wild resources compared to urbanized societies that are dedicated to industry or the provision of services [28]. For instance, Ruan-Soto et al. [29] found in Chiapas that the most mycophilous people were those who were engaged in activities in which they had greater contact with the environment. In this case, the peasants were the ones who had the greatest contact with mushrooms and other wild resources, which leads them to possess more complex knowledge about these organisms and not only about those that have a use [29]. Therefore, the insertion of new economic activities in which it is unnecessary to contact with nature could cause the relegation of local knowledge by other knowledge imposed by a hegemonic culture, and the cessation of traditional practices such as forest management, harvesting, and agriculture [30]. Consequently, the accumulation and acquisition of TEK that is promoted from traditional rural practices could be replaced by new practices and a western and urbanized lifestyle that avoids contact with nature [31, 32].

Regarding education, the formal school has been considered responsible for the loss of native languages in young generations, limiting the transmission of indigenous knowledge [33]. In some cases, formal education influences the loss of traditional knowledge, since tends to introduce arbitrariness to the acquaintance of society, dictating which knowledge is valid, disconnecting students from the environment, and devaluing local knowledge [34]. In macromycetes, not only does the number of known species of mushrooms decrease considerably in people with higher level of formal education [17], but knowledge about local taxonomy is affected by the lack of recognition criteria and traditional names [35].

It has been seen that age can also be an important factor, for example younger students are more receptive and curious about local knowledge than older students [36]. And in a community, the younger people usually have less knowledge than adults because the accumulation of knowledge is a process that takes place throughout a person's life [37]. According to Zent [38], the age at which most traditional knowledge is acquired exponentially are around 15 to 30 years old, after this point learning continues, but more slowly.

Furthermore, ethnomycological studies that have addressed TMK have generally done so from a qualitative approach [27, 28, 39, 40], and the few researches aimed at quantifying TMK, to date, have only considered the number of species mentioned and the local names [41–45]. Currently, work has focused on assessing the cultural significance of the species of edible and toxic fungi [46–49], but not the TMK holistically. For this, various questions arise: How can traditional knowledge be quantified? Is TMK affected by cultural and social changes? How do different sociocultural factors such as ethnic affiliation, migration, occupation, education, and age affect a person's TMK? What are the factors that influence a person to have a higher or lower TMK?

Our objectives were to propose indicators that would allow us to quantify the TMK and determine which are the social and cultural factors that can affect TMK among Wixaritari and Mestizos of Tlaltenango and Villa Guerrero. For this, we proposed 12 indicators with which TMK can be quantified. We carried out this work in two mycophilic sites, with high migration rates, in which an Indigenous group, such as Wixarika, and Mestizo people cohabit. One of these sites has had rapid development so that economic activities have diversified in a short time and the traditional lifestyle has been replaced by a modern one, while in the other site, the population continues to use wild products and economic activities are related to the field. We tested the hypothesis that Wixaritari adult persons who work in the field, with low level of education, and who have not migrated have the greatest TMK. We do not consider the gender factor because in other research carried out in these sites [18, 45], it has been observed that gender has no influence on the relationship between people and fungi.

## Methods

### Study area

**Fig. 1:**
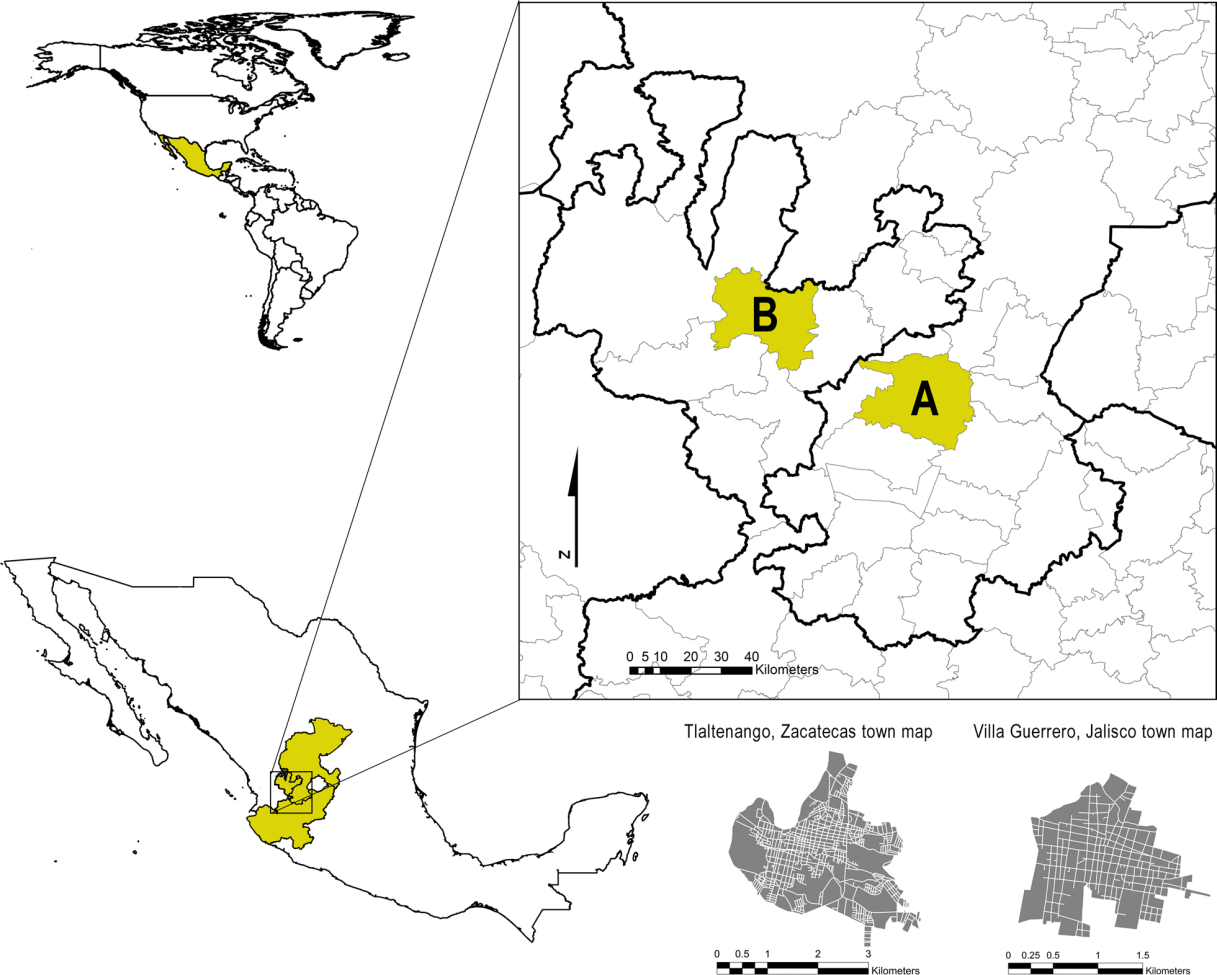
Map of studied sites. **A** municipality of Tlaltenango de Sánchez Román, Zacatecas; **B** municipality of Villa Guerrero, Jalisco

Tlaltenango de Sánchez Román is a municipality in the southwest of Zacatecas (Fig. 1), categorized by the Mexican institutions as a municipality with medium urbanization and a low degree of marginalization [50]. More information about the municipality is presented in Table 1. Only 4.5% of the total population was illiterate, 60.1% finished only basic education, 19.1% had upper secondary education, and 16.1% finished college [50]. The 69.66% of men and 30.34% of women were economically active, about 50% of the population was engaged in secondary and tertiary economic activities, and the main economic source was commercial activities [50] (Table 1).

**Table 1:** Ecological and sociodemographic characteristics of the study sites [3, 50]

Site	Tlaltenango	Villa Guerrero
Altitude m a.s.l	1600–2900	980–2360
Climate	Sub-humid and semi-warm sob-humid, rains in summer, temperature − 3 to 32 °C, precipitation 700–1000 mm	Semi-warm semi-humid and semi-warm semi-dry, average annual temperature 18.7 °C, average annual precipitation 700 mm
Vegetation	Sub-humid and semi-warm sob-humid, rains in summer, temperature − 3 to 32 °C, precipitation 700–1000 mm	High areas: pine-oak forests Low areas: subtropical scrub and grasslands
Inhabitants	26,748 (98%) Mestizos 554 (2%) Wixaritari 27,302 total*	5298 (94%) Mestizos 340 (6%) Wixarika 5638 total*
Economic activities	Secondary and tertiary (commercial activities and services)	Primary (agriculture and livestock)
Level of urbanization*	High	Low
Migration rate*	Low	High
Poverty level*	Low	High

*Until 2021[50]

Villa Guerrero is located in the north zone of Jalisco, Mexico, where the 47.7% of its surface is mountainous with slopes greater than 15° [51]. More information about the municipality is presented in Table 1. The level of education of the people in the municipality until 2015 was classified as low; 1,786 inhabitants were registered as illiterate, 68% had basic studies, and only 10% reached the upper secondary education. Therefore, it was classified as having a high level of marginalization especially the majority of the Wixarika population, and with a high migratory intensity and a low degree of connectivity on roads and highways. Its economy was based on agriculture and livestock; 72% of the population was engaged in agricultural activities, but not exclusively. Of the total population, 42.86% were engaged in commerce and 38.87% in tertiary activities. In the municipality, 64.6% of the inhabitants were in a situation of multidimensional poverty and only 13.6% had access to adequate food [51]. The inhabitants conserved the use of wild resources in daily life and Wixaritari were highly dependent on wild resources for their subsistence, and both, the Mestizos and the Wixaritari have a great taste for wild edible mushrooms [18].

### Sample selection and data collection

Before starting the fieldwork and data collection, we requested permits to carry out the interviews and publish the results. The purpose of the work was explained to the municipal political authorities and to each person interviewed, all this following the guidelines of the Code of Ethics of the Latin American Society of Ethnobiology [52].

We designed a format for conducting semi-structured interviews [53], which consisted of a first part with questions to collect sociocultural data such as cultural group, economic activity, age, formal education, language, origin, and whether the interviewee migrated at any time of their life. For the second part of the interview, the indicators shown in Table 2 were proposed based on ethnomycological studies that have to do with the ethnomycological knowledge of different societies and places. Each of these indicators was represented by a question. We had considered including the use of medicinal mushrooms as an indicator; however, it was already known from previous studies [18, 45], as in this fieldwork, that the use of medicinal mushrooms was non-existent in the region, so this point was not considered. Elsewhere, the use of mushrooms as medicine is a common practice, so in those cases the number of known medicinal mushrooms [54], attributed properties, and the methods of consumption or use should be included as indicators.

**Table 2:** Indicators and questions of the semi-structured interviews proposed to evaluate the ethnomycological knowledge in Tlaltenango, Zacatecas and Villa Guerrero, Jalisco, Mexico

Indicator	Question*	Score**	References
1. Named mushroom species in Wixarika or Spanish	What mushrooms do you know?	0–X	[2, 8, 31, 55–64]
2. Mushroom recognized species	In this photograph, which mushrooms do you recognize?	0–30	[8, 60, 65, 66]
3. Taxonomic finesse (knowledge about the morphological characteristics that help people to differentiate ethnotaxa)	How do you recognize an edible mushroom from a toxic one?	0–2	[25, 35]
4. Phenological knowledge about mushrooms	When does the sp_n_ grow?	0–2	[41, 58, 61, 67–69]
5. Ethnoecological knowledge about mushrooms (function of mushrooms in ecosystems, what it grows on, where it grows, and plant association)	Where does the sp_n_ grow?	0–2	[58, 61, 63, 70–73]
6. Knowledge about edible mushrooms	Which edible mushrooms do you know?	0–X	[17, 18, 61, 74–76]
7. Recipes and cooking methods	How do you cook the sp_n_?	0–2	[41, 58, 77]
8. Preservation methods	Do you keep mushrooms to eat in the dry season? How?	0–2	[17, 18, 58, 78, 79]
9. Knowledge of the nutritional contribution	What properties do mushrooms have as food?	0–2	[46, 80–82]
10. Uses of wild mushrooms	What are mushrooms for?	0–2	[17, 18, 41, 58, 61, 83, 84]
11. Propagation or promotion (techniques used to promote the growth of wild mushrooms)	Can you do something to get more mushrooms?	0–2	[18, 58, 85–89]
12. Knowledge about toxic mushrooms	Which toxic mushrooms do you know?	0–X	[18, 41, 49, 61, 90–93]

*sp_n_ refers to each of the mentioned or recognized species or ethnotaxa

** Score 0–X was coded as the total number of mentions. In the score from 0–2, 0 corresponds to a negative answer, 1 to a response on mushrooms in general, and 2 when the answer was specific to an ethnotaxon

The collection of images of basidiomycetes that were part of the visual stimuli used in indicator 2 was made from the works of Haro-Luna et al. [18, 44, 45], who collected the mushrooms known and used by the people of both municipalities. The visual stimuli consisted in 30 color photographs of 30 species of mushrooms taken in situ, showing all angles and characteristics of each fungus, printed at 11 × 17″ size (list of species in Attachment 1).

We applied these semi-structured interviews from July 2018 to May 2021. It is important to mention that in 2020 interviews were only conducted in January and February due to the COVID-19 contingency. We interviewed 150 people from Tlaltenango and 150 from Villa Guerrero, all older than 15 years of age. The people were chosen randomly, plotting quadrants on the maps of the study sites, and generating random coordinates in Excel 365. This was to cover the entire extension of the town, and one person from each address drawn was interviewed. The results of the interviews were captured in a database in the Excel 365 program, in which the rows correspond to each interviewee and the columns to each indicator evaluated here.

**Fig. 2:**
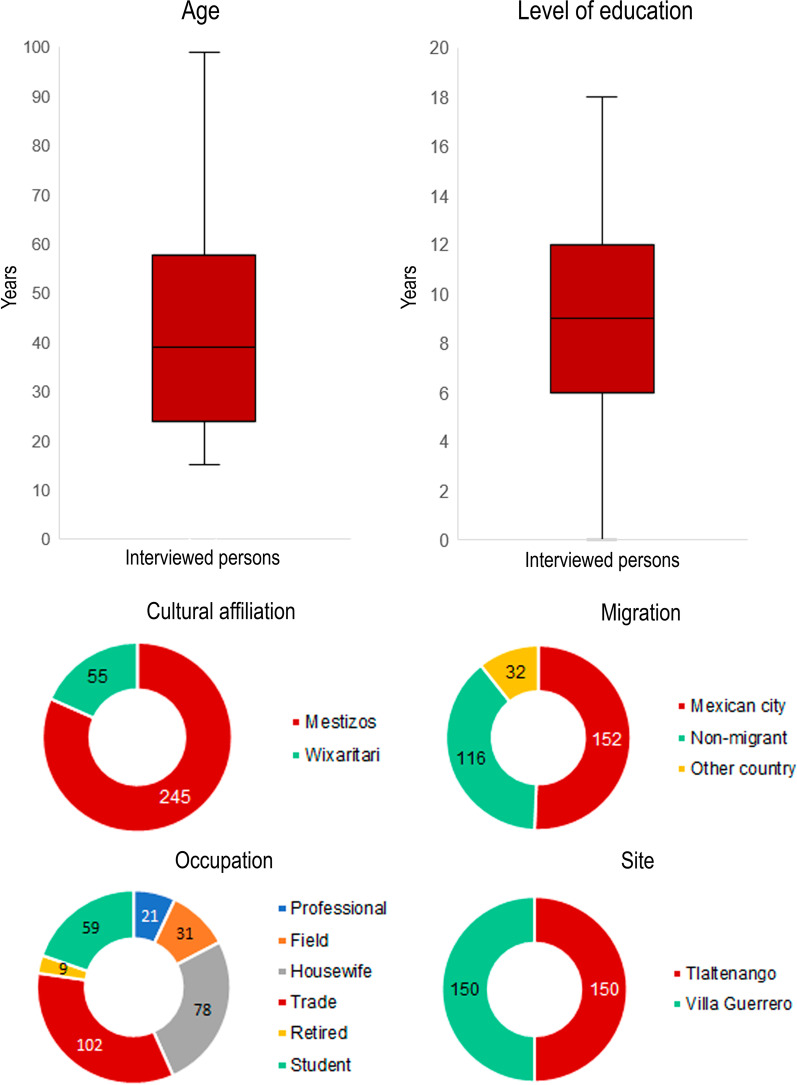
Interviewee's socioeconomic data

A principal components analysis (PCA) was performed with this matrix. The significance of the groupings obtained by PCA was tested with a discriminant function analysis (DFA). These analyses were performed using RStudio 1.4. In the DFA, we used three groups, two that were found in the PCA, separated by the main component one, and the third group was represented by Mestizo people with origin in rural areas since we expected that due to their place of origin, they would have a greater contact with the biota of the place where they grew up. We interviewed 192 women and 108 men. Of the total number of interviewees (300), the most representative occupations were trade and housewife (Fig. 2). Regarding the distribution of schooling, 26% of the interviewees have nine years of study, 15% have six years of studies, 13.33% have 12 years of studies, 0.6% have 18 years of studies, and 13% did not study (see Fig. 2).

## Results

**Fig. 3:**
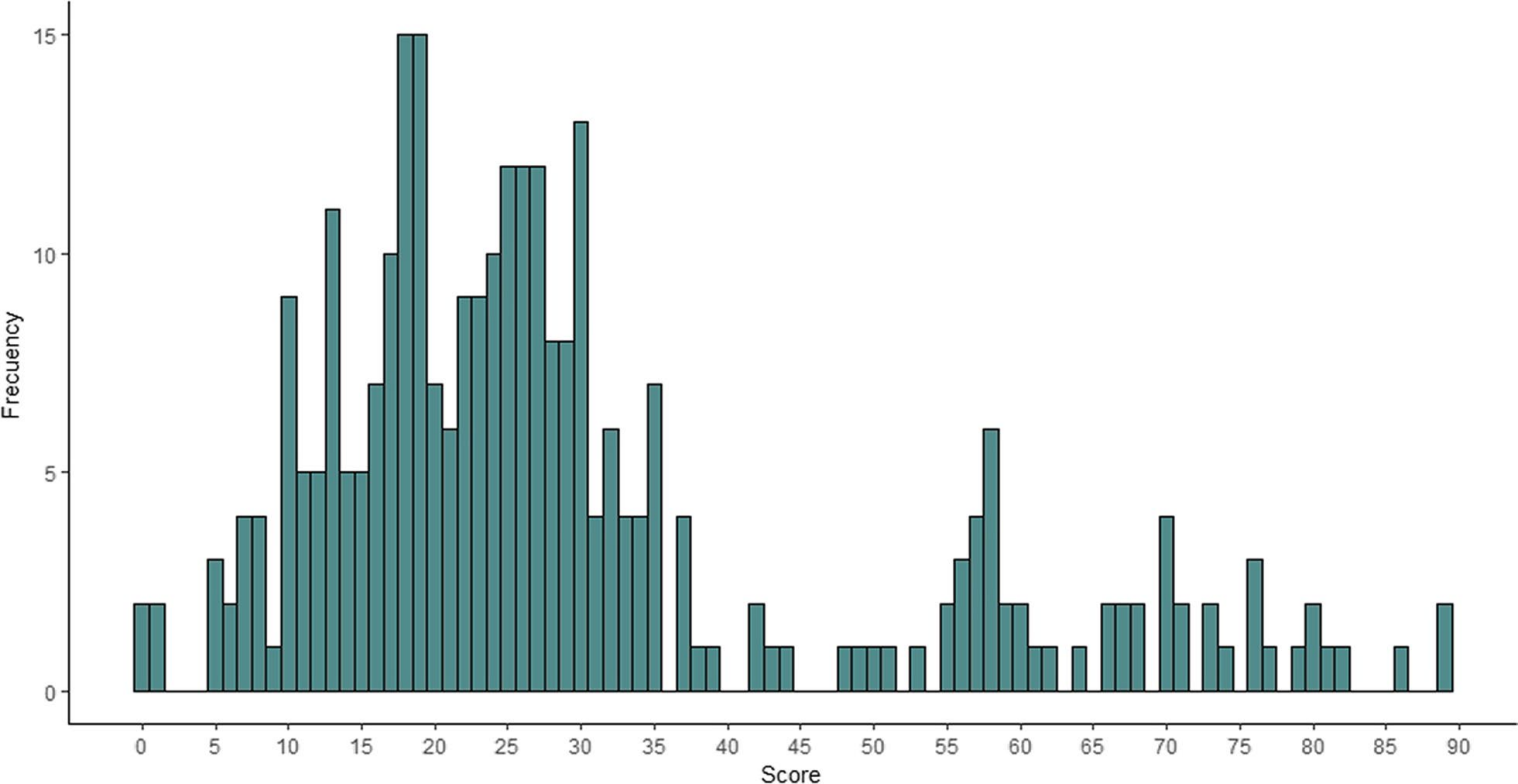
Frequency distribution graph of the global score of the people interviewed in Tlaltenango, Zacatecas, and Villa Guerrero, Jalisco, Mexico

In this study, the Wixaritari obtained a higher score than the Mestizos interviewed in relation with the TMK. The scores obtained in the responses on the ethnomycological knowledge ranged from 0 to 89. Most of the scores were between 19 and 28 points (st. dev. 19.07) (Fig. 3). The lowest scores (0–1 point) were obtained by non-migrant, Mestizo people who were between 35 and 75 years old, three men and a woman, with 9 to 16 years of study from Tlaltenango, Zacatecas. They were an engineer, a housewife, and two merchants. The people who obtained the highest score (86–89 points) were two Wixaritari women and a Wixaritari man from a rural community who migrated to Tlaltenango and Villa Guerrero for work. They were between 47 and 54 years old, studied from 0 to 6 years, and their occupations were craftswoman, day laborer, and merchant.

## Cultural affiliation, migration, and occupation

**Fig. 4:**
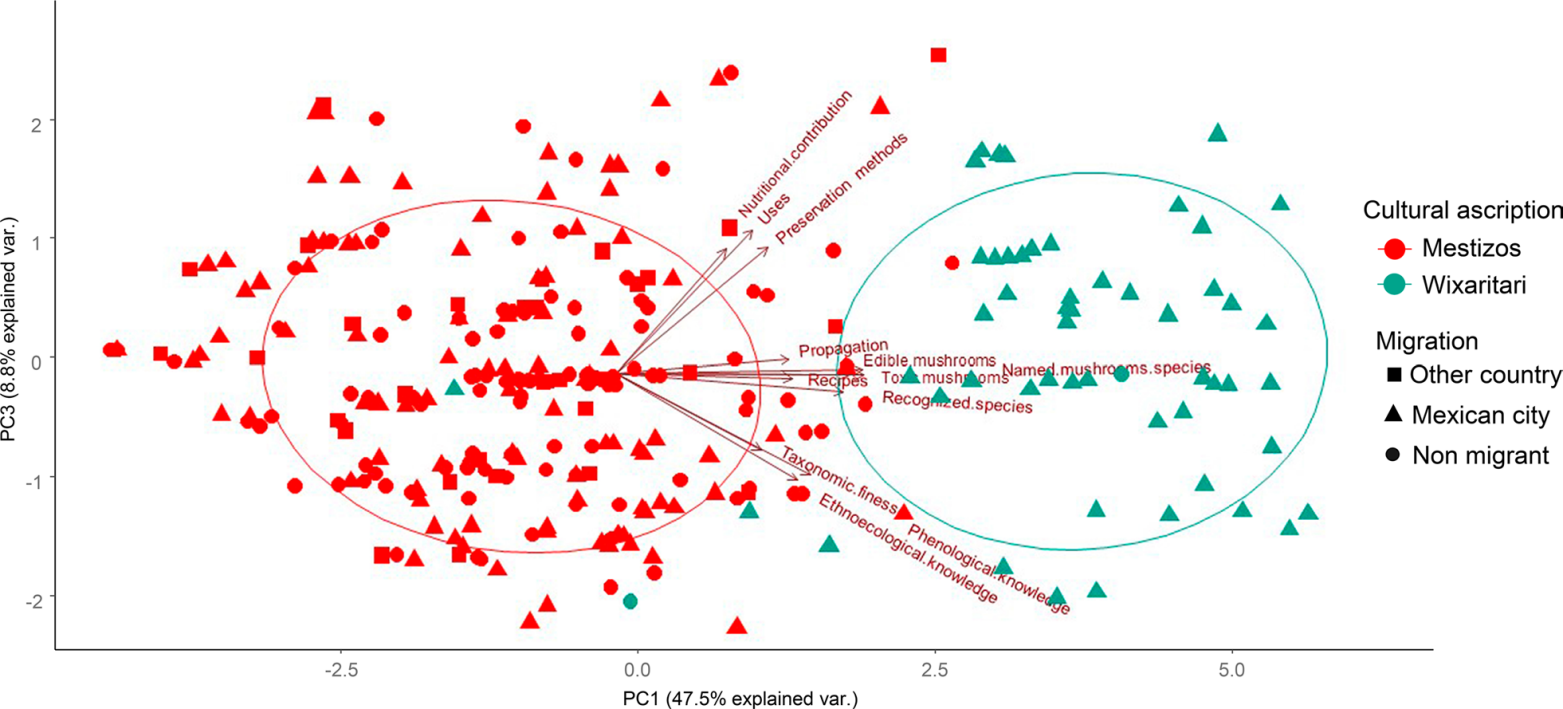
Principal components analysis of cultural ascription and migratory status of the interviewees in relation to mushrooms in Tlaltenango, Zacatecas, and Villa Guerrero, Jalisco, Mexico

The multivariate analyzes, specifically the PCA test, separated the interviewees into two groups according to the cultural affiliation of the people (Fig. 4). Despite what was expected, people were not separated by their economic activity, age, formal education, origin from rural or urban areas, or migration. As can be seen in Fig. 4, people who migrated within Mexico to urban places and those who migrated abroad were not separated from non-migrants. Likewise, those who dedicated themselves to economic activities in which there was no contact with nature were not separated from those who dedicated themselves to rural activities such as agriculture and livestock.

The principal component 1, which explained 47.52% of the variation (Table 3), was represented by the knowledge about edible mushrooms, named mushroom species, knowledge of toxic species, and mushroom recognized species (Table 4). This component separated the Mestizos from the Wixaritari. The principal component 2 explained 10.13% of the variation (Table 3) and was represented by the indicators: knowledge of nutritional contribution and taxonomic finesse (Table 4). The principal component 3 explained 8.76% of the variation (Table 3) and was represented by the indicators referring to the uses of wild mushrooms and preservation methods (Table 4).

**Table 3:** Percentage of variation explained in the first three components of the principal components analysis that assessed ethnomycological knowledge in Tlaltenango, Zacatecas, and Villa Guerrero, Jalisco, Mexico

Principal components	Eigenvalues	Percentage	% Accumulated
1	5.70	47.52	47.52
2	1.21	10.13	57.65
3	1.05	8.76	66.48

**Table 4:** Eigenvectors of the indicators used in the ethnomycological knowledge evaluation in Tlaltenango, Zacatecas, and Villa Guerrero, Jalisco, Mexico

Indicator	Principal components		
	1	2	3
Named mushroom species	**0.372**	− 0.285	0.011
Recognized species of mushrooms	0.344	− 0.178	− 0.064
Taxonomic finesse	0.221	**0.405**	− 0.270
Phenological knowledge about mushrooms	0.294	0.259	− 0.359
Ethnoecological knowledge about mushrooms	0.270	0.314	− 0.379
Knowledge about edible mushrooms	**0.375**	− 0.276	− 0.001
Recipes	0.267	0.098	− 0.015
Preservation methods	0.229	− 0.091	**0.444**
Knowledge of the nutritional contribution	0.167	**0.551**	0.439
Uses of wild mushrooms	0.206	0.229	**0.505**
Propagation	0.262	− 0.051	0.049
Knowledge about toxic mushrooms	**0.354**	− 0.319	− 0.001

The highest values in each of the principal components appear in bold

**Fig. 5:**
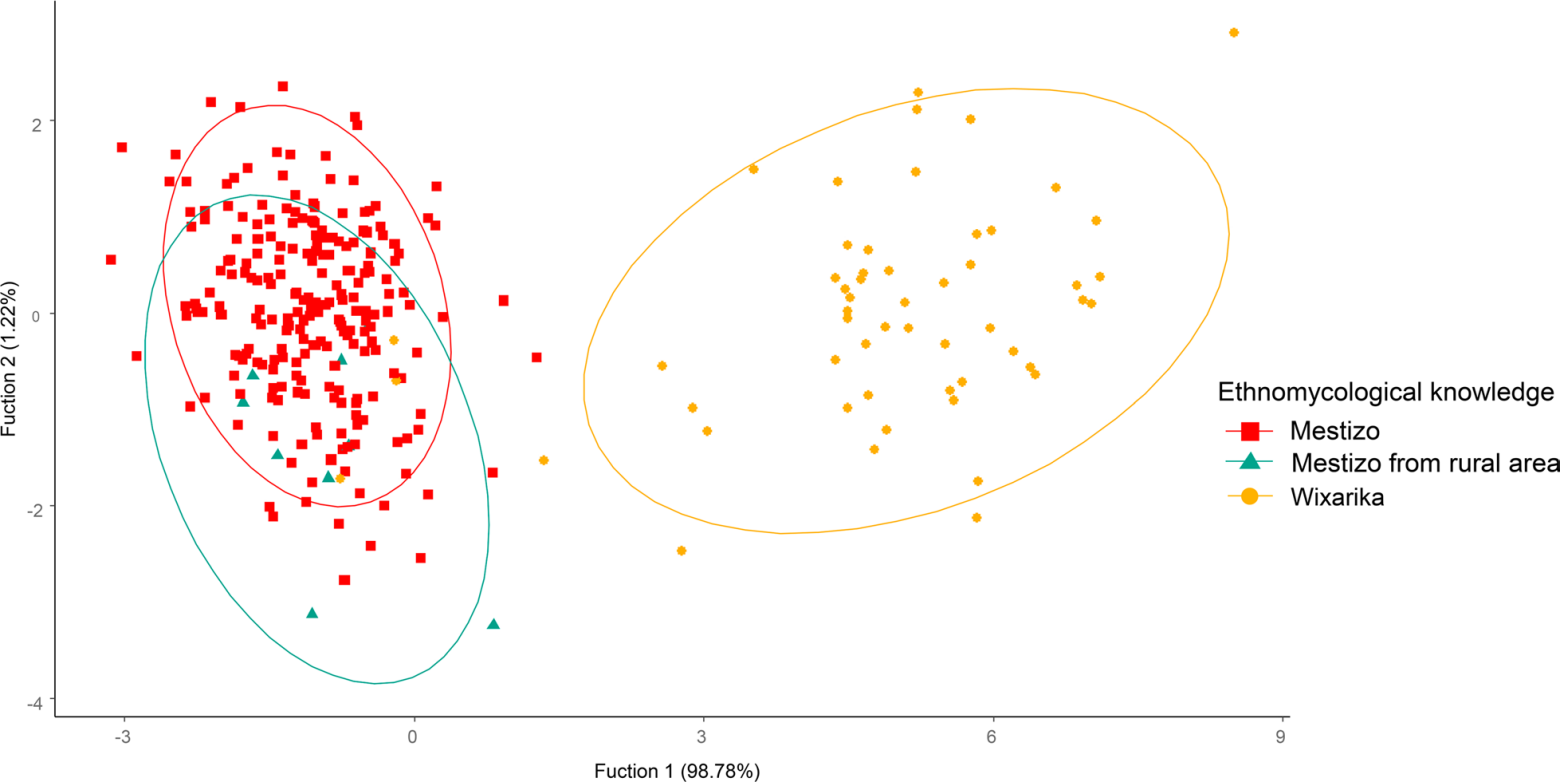
Discriminant function analysis according to the ethnomycological knowledge in Tlaltenango, Zacatecas, and Villa Guerrero, Jalisco, Mexico

DFA helped us to corroborate statistically the groups formed in the PCA (Tables 5 and 6). The first discriminating function separated the Wixarika from the Mestizo people. The most relevant indicators in the first discriminant function were propagation, knowledge of nutritional contribution, and knowledge about toxic fungi (Table 7). While the second function did not totally separate the Mestizos originated from a rural community, it was represented by the indicators ethnoecological knowledge about fungi and preservation methods (see Fig. 5 and Table 7).

**Table 5:** Discriminant function analysis of the ethnomycological knowledge evaluation in Tlaltenango, Zacatecas, and Villa Guerrero, Jalisco, Mexico

Discriminant function	Eigenvalues	Relative percentage	Canonical correlation	
1	5.412	96.333	0.918	
2	0.067	3.666	0.250	

Derived functions	Wilks Lambda	Chi square	d.f	Sig. level
1	0.146	560.614	24	0.000
2	0.937	18.913	11	0.062

**Table 6:** Classification of people according to their ethnomycological knowledge by the discriminant function analysis in Tlaltenango, Zacatecas, and Villa Guerrero, Jalisco, Mexico

	Predicted groups							
	1		2		3		Total	
Actual groups	Num	%	Num	%	Num	%	Num	%
Mestizo	236	100	7	77.77	4	7.27	236	100
Mestizo from rural area	0	0	2	22.22	0	0	9	100
Wixarika	0	0	0	0	51	92.72	55	100

**Table 7:** Coefficients of linear discriminants of the 11 indicators used to evaluate the ethnomycological knowledge in Tlaltenango, Zacatecas, and Villa Guerrero, Jalisco, Mexico

	Discriminant value	
	LD1	LD2
Named mushroom species	0.148	− 0.120
Recognized species of mushrooms	0.004	− 0.076
Taxonomic finesse	− 0.421	− **0.895**
Phenological knowledge about mushrooms	0.033	0.086
Ethnoecological knowledge about mushrooms	− 0.028	− **1.012**
Knowledge about edible mushrooms	0.229	0.420
Recipes	− 0.085	0.284
Preservation methods	0.485	− **0.688**
Knowledge of the nutritional contribution	− **0.564**	0.626
Uses of wild mushrooms	0.138	− 0.053
Propagation methods	− **0.988**	− 0.076
Knowledge about toxic mushrooms	**0.552**	0.077

The highest or lowest values in each of the main components appear in bold

According to the classification of people (Table 6), 100% of the Mestizos interviewed had shared knowledge. The 22.22% of the Mestizos with an origin in rural areas had particular knowledge, and of them, 77.77% had shared knowledge with the rest of the Mestizo people. Of the Wixaritari, 92.72% shared knowledge and only 7.27% had less knowledge, similar to that of the Mestizos.

As shown by the DFA (Table 6 and Fig. 5), four Wixaritari people did not behave like the rest. They were four female students, with 11 years of study, who migrated to a big city in the country to study, three of them from Tlaltenango, Zacatecas, and one from Villa Guerrero, Jalisco.

## Level of education and age

**Fig. 6:**
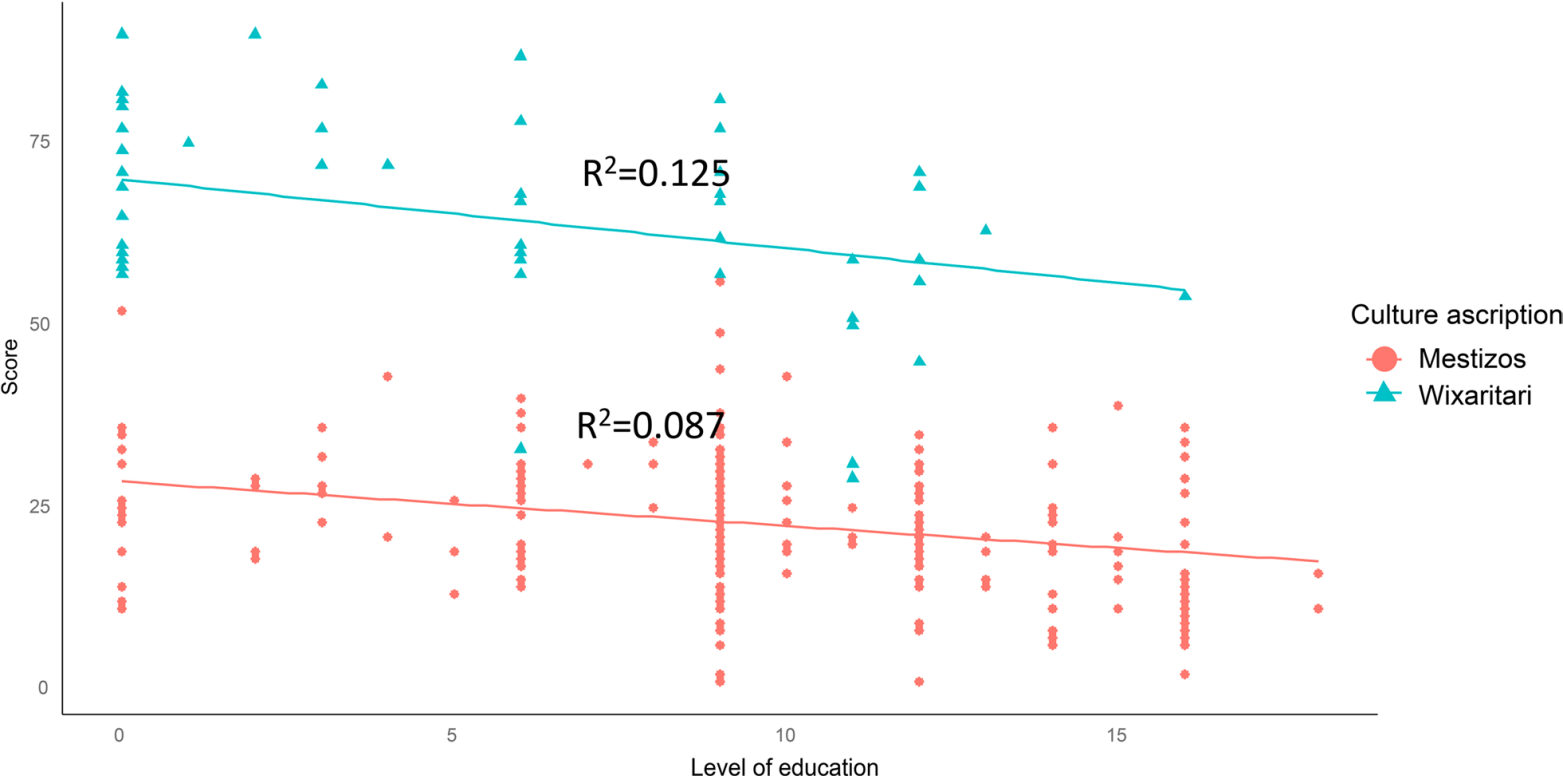
Lineal regression of traditional mycological knowledge score by level of education of people interviewed in Tlaltenango, Zacatecas, and Villa Guerrero, Jalisco, Mexico

**Fig. 7:**
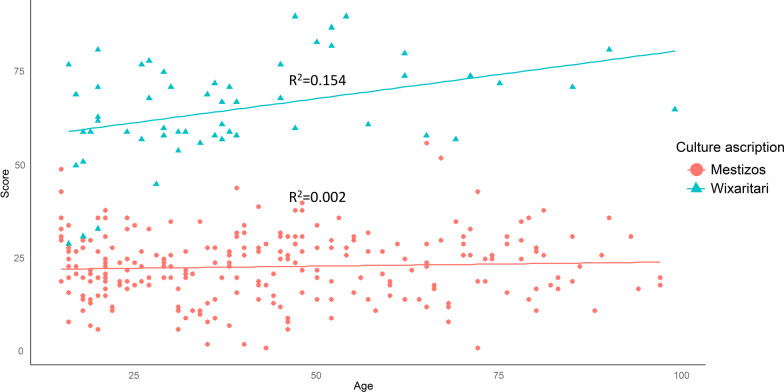
Lineal regression of traditional mycological knowledge score by age of people interviewed in Tlaltenango, Zacatecas, and Villa Guerrero, Jalisco, Mexico

We did not find a correlation between the age nor level of education of people and the traditional knowledge (Figs. 6 and 7). The correlation between the TMK score and the level of schooling was negative; however, the R^2^ value showed that it was not significant (0.125, 0.080) (Fig. 6). Likewise, although there was a positive trend between TMK and the age of the Wixaritari, the value of *R*^2^ (0.154, 0.002) showed that it was not significant. Meanwhile, the correlation of the traditional knowledge of the Mestizos was null (Fig. 6).

## Discussion

In the population considered in this study, the determining factor for a person to have greater TMK was ethnic affiliation. This coincides with Molnár and Babai [94], who proposed that the main TEK holders are indigenous groups with direct ties to the ecosystems, who create and maintain traditional knowledge through active use. This, according to Gosh and Sahoo [95], is because the communities have developed this knowledge about the biological diversity around them to make use of the greatest possible range of resources.

Contrary to expectations, we verified that the sociocultural factors that had been previously observed to influence the loss of ethnobiological knowledge [5, 9–13], such as age, migration, occupation, and level of education, were not relevant in this study in the TMK. The use of wild resources, the cultural roots that prevail despite social changes and modernity, as well as the importance of mushrooms as a food resource in these societies helped the prevalence of this knowledge, which coincides with Berkes et al. [1], who postulated that TEK prevails in societies that have given continuity to the use of resources throughout their history. In addition, people appreciate this knowledge as it was passed down to them from their parents and grandparents. It does not matter whether a person loses contact with their place of origin due to migration or their occupation, or their age, or years of study, provided that traditional knowledge is valued, it will remain alive as long as the elders transmit knowledge to the younger generations, who accept it and recognize the importance of this knowledge. Also, we must emphasize the importance of regional markets to maintain knowledge about traditional medicines and foods in Mexico [96]; for example, in this study it was seen that in an urbanized place like Tlaltenango, the sale of wild products has helped to keep alive the traditional diet that will be maintained if there is a demand and sale of wild mushrooms and other non-timber products.

In this study, people who emigrated abroad or to other cities within the country knew the same as the rest of the people of the same cultural group. Migration is usually associated with cultural changes and interference in the transmission of TEK, not only due to the loss of contact with resources, but also because the impact of the dominant societies reduces interest in preserving beliefs, practices, and knowledge [97, 98]. However, the people of Tlaltenango and Villa Guerrero preserved their knowledge, since they actively seek to have contact with the resources present in their place of origin despite the distance. The same phenomena have been reported with food and medicinal wild resources because migrants have the need to maintain their alimentary costumes and elements of their traditional medical practices [19, 99].

**Fig. 8:**
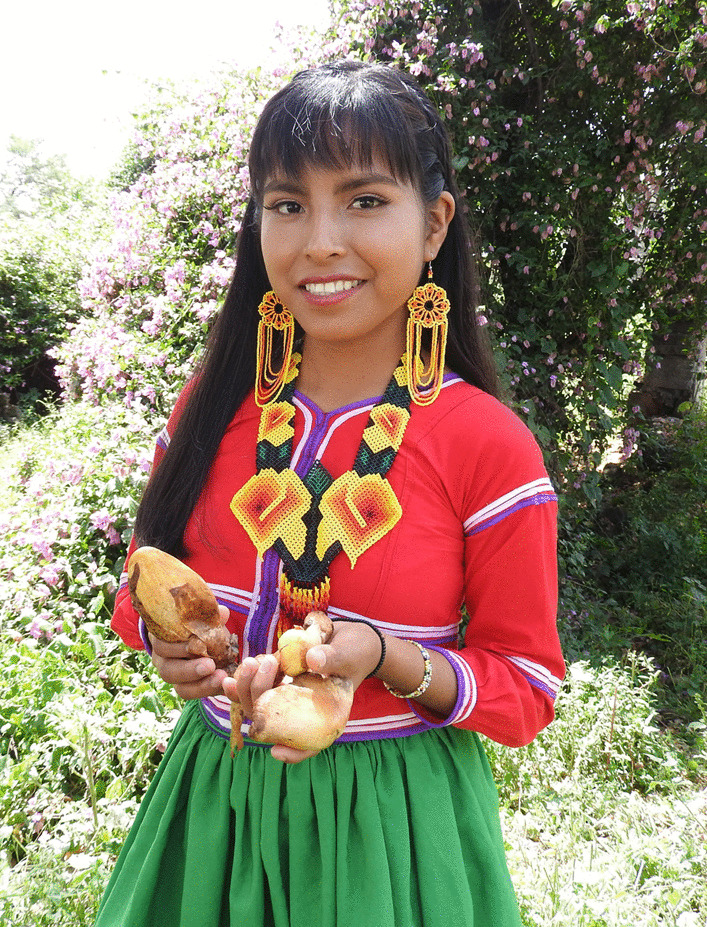
Sandra, a Wixarika woman who emigrated to Villa Guerrero holding *Volvariella bombycina*, one of the most culturally important edible mushrooms. Photograph by Mara Ximena Haro-Luna, August 19, 2019

In this case, Wixarika were the people with the highest scores in the indicators of TMK, and interestingly, they kept their knowledge, despite having migrated far from the forests where they used to collect mushrooms and having a different lifestyle than those who still live in the communities within the mountains (Fig. 8). This differs from what had been reported so far, in that the people who maintain closeness or contact with the collection sites retained a greater knowledge [100]. In the case studied here, they have preserved their knowledge and traditions despite living in another environment. This happens because the Wixaritari have an ideology that has resisted colonization, imposition, and hegemonic domination, so they have strong and conservative traditions and knowledge [101]. Therefore, this same phenomenon could occur in other ethnic groups with similar ideology resistance.

**Fig. 9:**
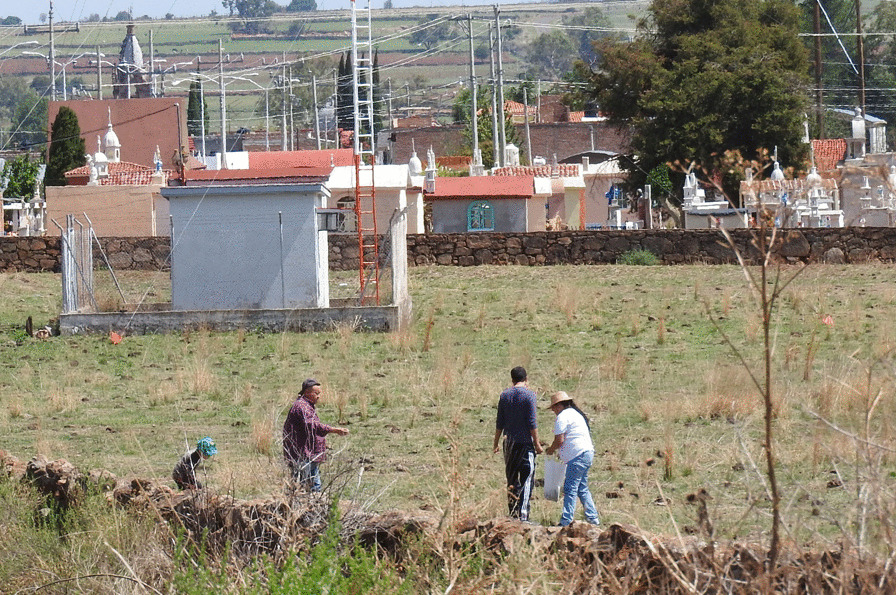
Family from Villa Guerrero, Jalisco harvesting *Agaricus campestris*. Photograph by Mara Ximena Haro-Luna, July 4, 2019

Contrary to expectations, the Mestizos who grew up in rural areas with greater contact with the biota did not have significantly different knowledge with respect to those who grew up in urbanized areas. In ecological ethnomycology, it is considered that the cultural implications with respect to the biota, like the TMK, are an adaptive trait to the ecological conditions with which a human group has contact [102]. The knowledge of the Mestizos who participated in this study corresponded to cultural backgrounds, such as use, harvesting (Fig. 9), recipes, and traditions about some mushroom species that were important in the area like *Agaricus campestris* (Fig. 8), *Pleurotus djamor*, and *Volvariella bombycina*, but not with other species such as those that grow in the temperate forest.

The variables with the greatest weight in the PCA to separate the Wixaritari from the Mestizos were the knowledge of ethnotaxa of edible and toxic fungi, with the Wixaritari knowing a greater diversity of fungi. These results coincide with what was reported by Robles-García et al. [103], in Querétaro, where the Mestizos knew a lower diversity of fungi than the Otomí people, an Indigenous group. But it differs with Montoya et al. [104], who reported that in the Mestizo communities of La Malinche volcano an even greater diversity of mentions was recorded with respect to the Nahua and Otomí communities.

According to the DFA, the most relevant indicators that separated the Wixaritari from the Mestizos were the knowledge of propagation, knowledge about the nutritional contribution, and knowledge about toxic fungi. Regarding propagation, the Mestizos mentioned the existence of different methods to favor the growth of mushrooms, their knowledge being focused on lignicolous fungi, in which they cut dry trunks of Ochote (*Ipomea intrapilosa*), soak them and leave them to rot in the shade near a stream, so that fungi can grow. Similar practices have been reported in central Mexico [87]. On the other hand, Wixaritari mentioned that there were no procedures to promote mushroom growth and that their appearance only occurs thanks to the rain. Nevertheless, they mentioned actions to increase the possibility of finding mushrooms in the forest, such as not throwing garbage or not starting fires. This perception is justified, since it has been proven that the mycobiota of a forest change after a fire; especially the amount of ectomycorrhizal fungi decreases [105, 106], which are the ones that the Wixaritari prefer.

For the Wixaritari, mushrooms are a nutritious food, but they do not attribute special qualities. For them, mushrooms are part of their traditional diet such as corn, beans, chili, and other wild plants and animals. For them, mushrooms serve to relieve the hunger of people and animals, or to “fill the belly,” so they contribute to their food security [107], while the Mestizos, as in central Mexico and northern Jalisco [18, 47, 103], considered that the mushrooms are nutritious and healthy food because they do not contain pesticides or chemicals.

Nowadays, some Mestizos from urban areas know that mushrooms are an important source of protein because of the media and nutrition campaigns of the Mexican government. However, even though the information disseminated in these campaigns includes fungi in vegetable dishes, the people interviewed classified fungi as a different form of life than plants and animals using criteria such as ecology, phenology, and morphology, just as the Mestizos from central Mexico do [108]. To them, mushrooms differ from plants because only grow for a short time each year, grow from rotting leaves and wood, and cannot be transplanted.

The Wixaritari were the ones who had the greatest knowledge about the diversity of toxic and useless fungi, to which they also gave a name, thus this was the third indicator with the highest weight in the DFA. Their vast knowledge was due to the importance of mushrooms in their worldview [49]. For the Wixaritari, toxic mushrooms were very important as guardians of the forests and were the spirits of edible mushrooms [18], while for the Mestizos they were not important, so they remained in a residual category of organisms [109], which included mushrooms such as Hongo hierboso (from “enhierbar,” meaning give someone a toxic mushroom). This demonstrated two types of classifications, one cognitive by the Wixaritari and another utilitarian by the Mestizos [110]. However, Mestizo people who grew up or lived in rural areas mentioned specific names for toxic mushrooms, such as Hongo de sapo (Toad mushroom), Hongo de víbora (Snake mushroom), and Hongo rojo de pino (Red pine mushroom), in the last case for *Amanita muscaria* [45].

In the discriminant function 2, the indicator with the most weight was ethnoecological knowledge, which although it did not separate people who grew up in urban areas, yielded important answers. The ethnoecological knowledge in the study sites was linked to their traditional classification system. Mestizo people grouped mushrooms that grow in grasslands, *Agaricus campestris* and *Calvatia cyathiformis*, in the ethnotaxon Hongo de llano, which had great cultural importance in Tlaltenango, but not in Villa Guerrero. The Mestizos also mentioned Hongos de barranca (Canyon mushrooms), which were lignicolous fungi that grow on decomposing *Ipomea intrapilosa* trees (Ochotes), such as Hongo de ochote (*Volvariella bombycina*) and Oreja de ochote (*Pleurotus djamor*). Another of these Hongos de barranca was *Pleurotus djamor*, known as Hongo de cazahuate in Tlaltenango, the same name being reported in the state of Morelos [111]. Hongos de la sierra (Mushrooms of the mountains) included all the ectomycorrhizal and saprobic mushrooms of *Quercus* and *Pinus* forests, which were less mentioned by people who had less contact with this type of ecosystem, either due to their lifestyle or occupation.

Preservation method was an indicator included in the discriminant function 2. Wixaritari usually sun-dry their mushrooms, particularly *Pleurotus djamor*, to preserve them throughout the year [18]. A few people from Mestizo communities did the same [45]. In urban areas, ignorance of some preservation method predominated, and it was considered a highly perishable food, but 58 people also mentioned keeping them already cooked and frozen, and two adult people from Tlaltenango mentioned that they did not know how to preserve mushrooms, but they knew canned mushrooms. The acquisition of processed food products corresponds to an urban and globalized food model [112]; therefore, the consumption of canned mushrooms that were bought in supermarkets was more common in Tlaltenango than in Villa Guerrero.

Although the level of formal education is usually related to the loss of traditional knowledge [33, 34], we found no relationship between the level of formal schooling and the accumulation or loss of TMK. Although three Wixaritari who were young people and studied for nine to 11 years had a similar knowledge to Mestizos, i.e., less knowledge than other Wixaritari, there were other Wixaritari interviewed with 12–16 years of study who had high scores in the indicators of TMK. Some Wixaritari mentioned that their lack of knowledge was caused by personal disinterest or because they did not like the taste of mushrooms. According to Arjona-García et al. [113], this attitude responds to changes in lifestyle, social roles, and perceptions caused by urbanization and the substitution of the traditions.

**Fig. 10:**
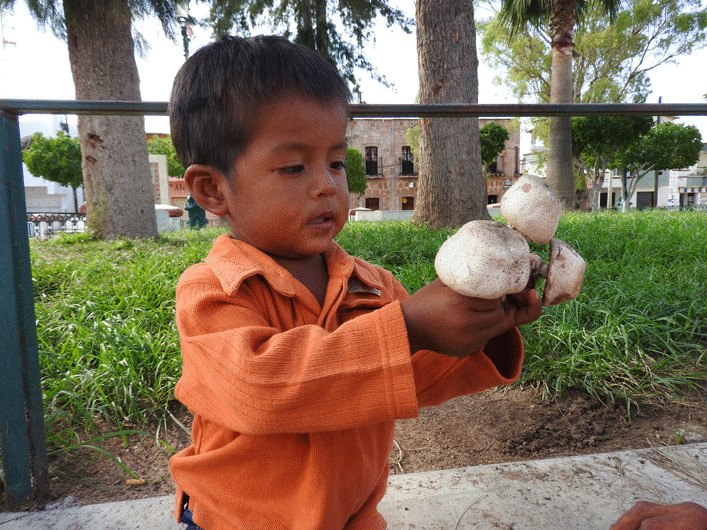
Wixarika child playing with non-edible mushrooms. Photograph by Mara Ximena Haro-Luna, July 29, 2019

Traditional knowledge is generally considered to continue to accumulate throughout a person's lifetime [114, 115]. Therefore, an older person in a community is expected to have more knowledge than younger ones [116]. However, we did not find a relationship between age and a person's level of traditional knowledge. This result has already been found in traditional knowledge about plants and animals [32, 117]. It can be explained because the acquisition of TMK probably occurs exponentially before the age of 15 [32, 118] (Fig. 10). In addition to that it must be considered that although they have a similar wealth of knowledge, their application in daily life and practical knowledge varies according to their age and the role they play in society [119].

## Conclusion

In this study, the cultural group to which a person belongs was one of the determining factors to know who has a greater TMK. Nevertheless, it should be considered that social and environmental changes may affect this knowledge, and thus, their influence should not be underestimated. TMK is complex, and many factors can affect its distribution in the population; however, it is shared by people who belong to a culture. In this case, the Wixaritari had strong cultural roots and despite changing their lifestyle they continued to feel pride in their cultural identity; this has helped them preserve their knowledge and elements of their culture. Otherwise, the Mestizos adapted to a modern lifestyle and urbanization [103], leaving aside the use and exploitation of wild products.

In this work, 12 indicators were proposed to evaluate TMK, which worked in the study areas and were appropriate to the zone. In subsequent studies in other areas, these indicators can be used, some could be changed and adapted to the circumstances, and others could be included. Likewise, it would be interesting to carry out a similar study considering children under 15 years of age. This would help to verify at what age most of the traditional mycological knowledge is acquired. In addition to the fact that Wixaritari children are exposed to a lifestyle different from that of Mestizo children, in which aspects of their worldview, culture, and traditions are instilled in them from a very young age.

As we observed in this work, TMK included different aspects. Although a person knows few fungi, he may have a wide knowledge about those species and a person who may know or recognize many species of fungi might not know anything about their ecology, phenology, edibility, etc. In addition, there is specific knowledge that must be taken with caution since it is exclusive to a small sector of the population, defined by the role that these people have within society, such as traditional doctors, shamans, or local wise person.

## Data Availability

Not applicable.
